# Perceptions of social norms around healthy and environmentally-friendly food choices: Linking the role of referent groups to behavior

**DOI:** 10.3389/fpsyg.2022.974830

**Published:** 2022-10-13

**Authors:** Elif Naz Çoker, Susan A. Jebb, Cristina Stewart, Michael Clark, Rachel Pechey

**Affiliations:** ^1^Nuffield Department of Primary Care Health Sciences, University of Oxford, Oxford, United Kingdom; ^2^Oxford Martin School, University of Oxford, Oxford, United Kingdom

**Keywords:** social norm, referent groups, food choice, norm and behavior congruence, sustainable food choice, healthy food choice, eating behavior

## Abstract

Referent groups can moderate the perception of social norms and individuals’ likelihood to model these norms in food choice contexts, including vegetable intake and reduced meat consumption. The present study investigated whether having a close vs. a distant social group as the referent changed perceptions of social norms around making healthy and eco-friendly food choices. It also assessed whether these changes were associated with a difference in the health and environmental impacts of food choice in a virtual grocery shopping task. A nationally representative sample of UK adults (*N* = 2,488) reported their perceptions of making healthy and eco-friendly food choices being the norm among people they share meals with (close referent group) and most people in the UK (distant referent group). The former was more commonly perceived to be making both healthy (Z = −12.0, *p* < 0.001) and eco-friendly (Z = −13.27, *p* < 0.001) food choices than the latter. Perceptions of norms referring to the close group were significantly associated with the environmental (β = −0.90, 95% CIs: −1.49, −0.28) and health (β = −0.38 *p* < 0.05, 95% CIs: −0.68, −0.08) impacts of participants’ food choices in a virtual shopping task. No such relationship was found for norms referring to the distant group for both environmental (β =0.43, *p* > 0.05, 95% CIs: −1.12, 0.25) and health (β = −0.06, *p* > 0.05, 95% CIs: −0.37, 0.25) impacts. Framing social norms around making healthy and eco-friendly food choices to refer to a close referent group may change their perceptions and ability to encourage sustainable and healthy food purchasing.

## Introduction

While general awareness about the importance of diet on public health and the environment has increased over the years, the EAT-Lancet Commission on Food, Planet and Health suggests that considerable changes need to be made to make our diets healthy and sustainable, in particular cutting down our consumption of meat and other animal proteins (Willett et al., 2019). Clark et al. (2019, 2020) report that foods associated with improved health (such as fruits, vegetables, legumes, nuts and whole grain cereals) are also among those that have the lowest environmental impact in terms of greenhouse gas emissions, loss of biodiversity, and excessive land and water use. Understanding the enablers and barriers behind eating behavior, and capitalizing on these to encourage better food choices for both humans and the planet is crucial for a sustainable future.

Individuals are influenced by the eating behaviors of others in social settings when making their own food choices (Robinson et al., 2014; Sharps and Robinson, 2016; Cheah et al., 2020). These social norms can be defined as a set of informal rules or standards for the behaviors of individuals that are generally agreed upon by the members of a social group (Fehr and Fischbacher, 2004). Social norms can serve as decisional heuristics, helping individuals identify what constitutes usual behavior (Cialdini and Goldstein, 2004; Brachem et al., 2019) in a variety of contexts, including food choices. Recent studies and systematic reviews have provided evidence that social norms can be significant influencers in guiding eating behavior (Robinson et al., 2014; Higgs, 2015; Raghoebar et al., 2019).

Referents are individuals or groups who are observed for norm signals that indicate accepted and desired behavior (Paluck and Shepherd, 2012). Individuals are inclined to behave coherently and consistently with the social group with which they identify (Childers and Rao, 1992). The social proximity of the referent group that sets the social norm to the individual could make the norm more salient and more likely to be followed. Perceived similarities between the individual and the referent group might help the individual to have a higher belief in their self-efficacy for engaging with the behavior or feel that their self-identity is congruent with the behavior (Higgs, 2015).

Social Identity Theory (Turner et al., 1987) suggests that stronger associations with a group makes an individual more likely to want to conform to the behaviors set out by said group. The influence of a norm can be increased if the individual finds the norm referent group relevant and proximate to their own identity, making the individual more likely to model the norm (Stok et al., 2012, 2016; Liu et al., 2019; Liu and Higgs, 2019). Individuals have more opportunities to exchange information with, observe the behavior of, and learn the judgments and values of their close social circles, which means that they can become more confident in their knowledge of social norms of their proximal referent groups. In the context of eating behavior, studies have found evidence that the referent group moderated the level of influence of social norms in the consumption or avoidance of certain food items, where individuals followed the norm when it was presented as coming from a group they identified with as opposed to an out-group (Stok et al., 2012; Robinson et al., 2014).

While current literature provides evidence that referent groups play a role in the perception of social norms in the context of food choices, existing studies have often relied on small sample sizes, focused on the intake or avoidance of certain food groups, and measured self-reported intentions rather than actual food purchase and consumption behaviors. The present study extends this research by recruiting a large and representative sample of the United Kingdom (UK) and examines the relationship between social norms and food purchasing using a virtual supermarket setting which provides a strong proxy for actual behavior. We examined how individuals’ perceptions of making healthy and environmentally friendly food choices as normative behaviors differed when the norm was framed to refer to a close (“people I share my meals with”) versus a distant (“most people in the UK”) referent group. Based on previous literature, we hypothesized the following:

*H1:* Perceptions of social norms will have predictive power on the total environmental and health impact of participants’ shopping baskets such that those who believe that it is the norm to make more environmentally friendly and healthy food choices will have shopping baskets with healthier and more environmentally sustainable impacts.

*H2:* Perceptions of social norms that refer to the close referent group will have a better predictive power on the total environmental impact of participants’ shopping baskets than norms that refer to the distant referent group.

## Materials and methods

### Procedure

**Figure 1:**
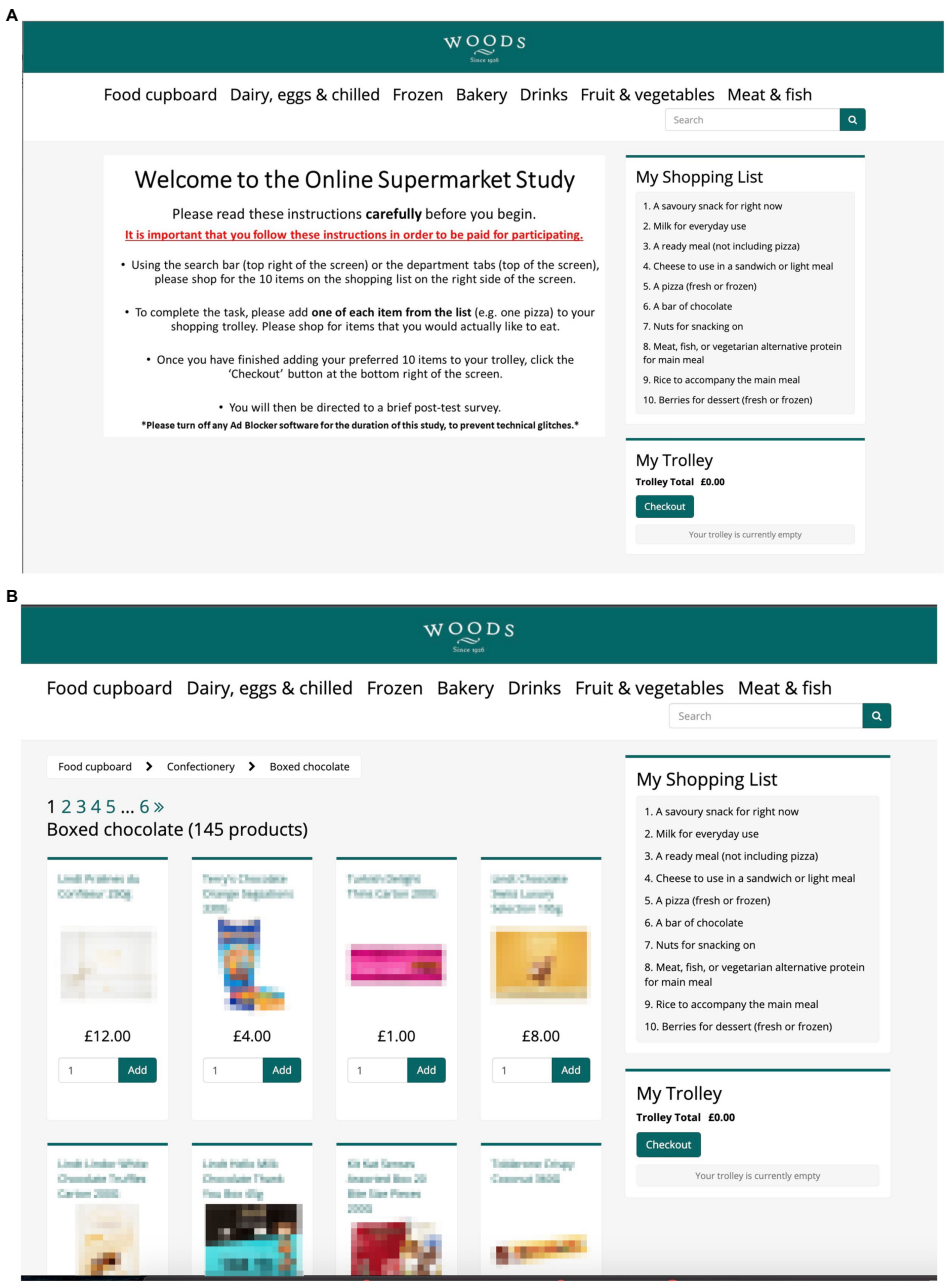
**(A)** Screenshot of the welcoming screen of the experimental online supermarket platform. **(B)** Screenshot of one of the product aisle pages of the experimental online supermarket platform.

Data for this study was collected as part of a post-task survey following a food labelling intervention study (Potter et al., 2022b). The study protocol was reviewed and approved by the University of Oxford’s Central University Research Ethics Committee [R65010/RE004]. As part of the intervention study, participants were directed to an online virtual supermarket platform (see Figures 1A,B) where they were asked to choose products corresponding to items on a shopping list prepared by the researchers (see Table 1). The platform was developed by the University of Oxford with the aim to emulate a real online supermarket as closely as possible, including 20,000 supermarket products extracted from a database of food and drinks that are available for purchase in six UK online supermarkets (Harrington et al., 2019). Participants are able to browse department, aisle, and shelves of products, see product size and nutritional information, add items to their trolley, and view a check out page. The platform has been previously used to test interventions and nudges such as product swaps to reduce salt and energy content (Forwood et al., 2015; Koutoukidis et al., 2019; Riches et al., 2019), labeling lower-energy and environmentally friendly products to increase their selection (Marty et al., 2020; Potter et al., 2022a), and including health, cost and social norm messages (Bunten et al., 2021) and has the advantage of creating key qualities of a grocery shopping environment such as large number of products, browsing locations within the store, labeling, and other marketing techniques that would be lacking in a laboratory setting and of manipulating these qualities which would not be feasible in most real-life shopping settings. Furthermore, “studies of nutritional labelling in experimental supermarkets have suggested that while the effect sizes in experimental studies may be larger than for actual purchases, the pattern of results is relatively consistent between these settings” (Crosetto et al., 2019; Howe et al., in press).

**Table 1:** Shopping list provided to participants in the online virtual supermarket task.

1.	A savoury snack for right now
2.	Milk for everyday use
3.	A ready meal
4.	Cheese to use in a sandwich or light meal
5.	A pizza (fresh or frozen)
6.	A bar of chocolate
7.	Nuts for snacking on
8.	Meat, fish, or vegetarian alternative protein for main meal
9.	Rice to accompany the main meal
10.	Berries for dessert (fresh or frozen)

Participants did not spend any real money, nor receive the products they chose but were asked to imagine they were shopping, and select things they would normally choose. Participants’ shopping baskets were assessed and assigned total health and environmental impact scores ranging from 0 (most healthy/environmentally friendly) to 100 (least healthy/environmentally friendly). The food labelling intervention study (Potter et al., 2022b) randomly allocated participants (*via* randomizer elements on Qualtrics, with five participants randomized into an intervention arm for every two randomized to the control group) to one of four intervention arms which either displayed labels on food items that indicated their environmental impact, health impact, both impacts, or a control arm with no labels.

After completing the virtual shopping task, participants were directed to a post-task survey where they provided information on their demographic characteristics and eating habits. At this time, they were also presented with four social norm statements (detailed in the measures sections below) and asked to state their level of agreement with each statement, which provided the data for the present study.

The present study and the food labelling intervention study were both pre-registered on the Open Science Framework (/osf.io/zg29d; https://osf.io/gkyds/).

### Participants

Participants were recruited through Dynata, an online market research firm, and the sample was nationally representative of the UK in terms of gender, age, education, and income. Participants were included if they met the criteria of being aged 18 years and above, based in the UK, able to read and understand English, willing and able to give informed consent, and having access to, and familiarity with, a computer and the Internet. Since the study required participants to make food choices that included meat and dairy, people following a vegetarian or vegan diet were excluded from participation. A total of 2,730 participants consented take part in the study and 2,488 completed the survey and provided demographic information, resulting in a 9.12% attrition rate.

The sample size was determined based on the effect size for the impact of environmental impact labelling on the environmental impact of food purchases (an absolute difference of 4%). As this was an add-on study, no formal power calculations were conducted for the research questions addressed here.

### Measures

#### Perceptions of social norms

Participants were asked to state their level of agreement with four statements on a 5-point Likert scale ranging from “strongly disagree” (1) to “strongly agree” (5). The statements differed in their context (environment or health) and referent group (distant or close) to assess perceived social norms around food choices:

1. “**Most people in the UK** will try to choose the food items that are better for the **environment.**” (environment, distant)
2. “**People who I share my meals** with will try to choose the food items that are better for the **environment**.” (environment, close)
3. “**Most people in the UK** will try to choose the food items that are better for their **health**.” (health, distant)
4. “**People who I share my meals with** will try to choose the food items that are better for their **health**.” (health, close)

The decision to choose the wording “People who I share my meals with” was reached following structured discussions among the research team. Keeping in mind that individuals might have different social and familial situations in which they might eat with family members, friends or housemates, significant others, or close colleagues, this wording was chosen to encompass all these probabilities and prompt participants to think about people they are socially close to with whom they eat together.

#### Shopping basket environmental impact scores

Environmental impact scores were calculated by linking ingredient lists with a publicly available global environmental Life Cycle Assessment database (Poore and Nemecek, 2018) and based on greenhouse gas emissions, water use, biodiversity loss and water pollution. Impacts for the four indicators were collapsed into a single product-level score (0–100; lowest-highest impact; most sustainable to least sustainable). These scores were derived by identifying the composition of each ingredient in a food product, and then estimating the composition of each ingredient in each food using prior known information from similar products, nutrition information for that product, and UK labelling regulations on how ingredients must be reported on packaging information. Each ingredient in a product was then classified into an appropriate category to correspond to categorizations on the environmental database, The estimated composition of food products and the environmental database, which provides estimates of the environmental impacts per 100 g of each food category, were then used in combination to estimate the environmental impact per 100 g of each product for the four environmental indicators listed above (Poore and Nemecek, 2018). The scores for these indicators were then aggregated into a single composite product-level score which range from 0 (lowest environmental impact; most environmentally sustainable) to 100 (highest environmental impact; least environmentally sustainable). Detailed information on calculating the environmental impact scores is described elsewhere (Clark et al., 2022; Potter et al., 2022b).

#### Shopping basket health impact scores

Health scores were calculated using the NutriScore method (Chantal et al., 2017) which considered the composition of “nutrients to limit” (e.g., sugars, saturated fats) and “nutrients to encourage” (e.g., proteins and fiber) which assigned a score each product from 0 (most nutritious) to 100 (least nutritious). Each product is given a NutriScore based on seven food components: energy, saturated fat, salt, sugar, fiber, protein, and the amount of fruits, vegetables, nuts, and some oils. Each of these seven components is given a score (scores for the first four components range from 0 to 10; scores for the last three components range from 0 to 5) against pre-set thresholds. In general, but with exceptions for certain types of foods (e.g., fats, cheese, etc.) and for drinks, the scores for the first four components are summed together (for a maximum of 40), and then the summed score of the last three components (a maximum of 15) are subtracted from the summed score of the first four components. This numeric score can therefore range from −15 to 40, with lower scores indicating a better nutrition quality. This numeric score was then scaled to range from 0 to 100, such that 0 indicates the best possible nutrition composition and 100 indicates the worst possible nutrition composition. The full procedure is again detailed in Potter et al. (2022b).

### Data analysis

The data was analyzed using STATA (16.1, StataCorp LLC). Average Likert scale ratings for each of the four social norm items were calculated. Differences between social norm item ratings were calculated using Wilcoxon signed-rank tests. Differences between environmental impact and health impact scores across levels of agreement with social norm statements were calculated using Kruskal–Wallis and Dunn’s post hoc tests to address non-normality of the data. Linear regressions using robust estimators of variance to overcome problems of heteroskedasticity and non-normal residuals were run to predict the environmental and health score of participants’ shopping baskets based on participants’ perceptions of social norms around making environmentally friendly and healthy food choices with respect to their close referent group and the general population in the first model. In a second model, intervention arm allocation was entered as a covariate. Data analysis deviated from pre-registered plans in that distant and close social norms models were not compared to each other, given there was no evidence that distant norms had predictive power on environmental and health impact scores.

## Results

### Participant demographics

2,481 out of 2,488 participants who completed the survey gave answers to the social norm questions. The age of the participants ranged from 18 to 69 (M = 41.66, SD = 13.29), 55.4% of the participants were female, 41.8% had received higher education (Bachelor’s degree or above), and 23.8% fell into a higher income bracket (£40,000 and above; see Table 2).

**Table 2:** Demographic characteristics of participants (*n* = 2,481).

Demographic categories	Frequency	Valid Percentage
Age (years)		
18–24 25–34 35–44 45–54 55+	344 478 552 609 747	12.6 17.5 20.2 22.3 27.4
Gender		
Female Male Not specified	1,377 1,111 242	55.4 44.6
Education		
1–4 GCSEs 5+ GCSEs or 1 A-level 2+ A-levels Bachelor’s degree Graduate degree Not specified	326 462 648 702 331 261	13.2 18.7 26.3 28.4 13.4
Income		
Less than £15,000 £15,000 – 24,999 £25,000-39,999 £40,000-75,000 Over £75,000 Not specified	638 520 588 436 109 439	27.9 22.7 25.7 19.0 4.8

### Shopping basket environmental impact scores

Participants’ shopping baskets had an average environmental impact score of 61.82 (S.D = 6.18), with the participant with the lowest impact basket having a score of 26.26 and the highest having 80.87.

### Shopping basket health impact scores

Participants’ shopping baskets had an average health impact score of 40.85 (S.D = 3.43), (S.D = 6.18), with the participant with the lowest impact basket having a score of 28.18 and the highest having 62.96.

### Perceptions of social norms based on referent group

**Figure 2:**
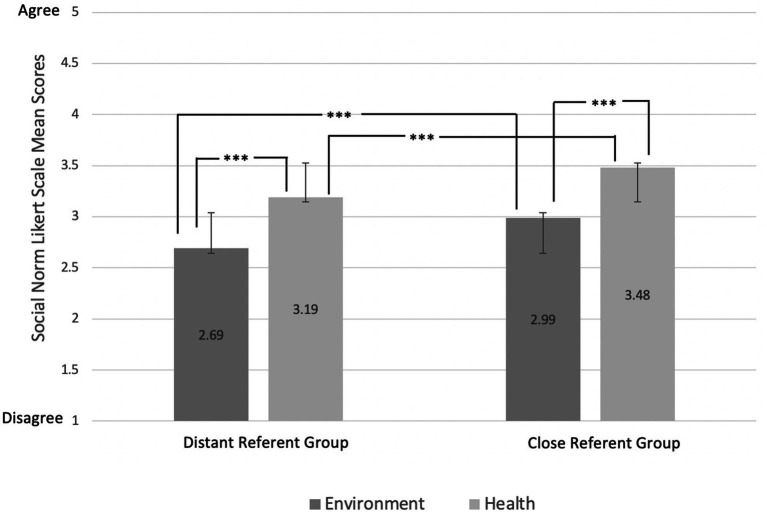
Means of responses to social norm items^*^ (1 = strongly disagree – 5 = strongly agree) (*** = *p* < 0.001). *“**Most people in the UK** will try to choose the food items that are better for the **environment**.” (environment, distant). “**People who I share my meals** with will try to choose the food items that are better for the **environment**.” (environment, close). “**Most people in the UK** will try to choose the food items that are better for their **health**.” (health, distant). “**People who I share my meals with** will try to choose the food items that are better for their **health**.” (health, close).

The means and standard deviations of the Likert scale ratings (possible range: 1–5) of all four social norm items are shown in Figure 2. Wilcoxon signed rank tests indicated that participants were more likely to perceive trying to make healthier food choices to be the norm compared to environmentally friendly choices, regardless of referring to distant (Z = −23.96, *p* < 0.001, r = 0.005) or proximal (Z = −23.84, *p* < 0.001, r = 0.0048) referent groups. With regards to the differences between referent groups, Wilcoxon tests indicated that participants perceived their proximal referent group to try to make both healthier (Z = −12.08, *p* < 0.001, r = 0.0024) and more environmentally friendly (Z = −13.27, *p* < 0.001, r = 0.0027) food choices compared to the UK general population (*N* = 2,481). Kruskal–Wallis tests indicated that there were no differences in responses to any of the social norm items across experimental conditions of the Potter et al. (2022a,b) study.

### Effects of perceptions of social norms on shopping basket environmental scores

**Figure 3:**
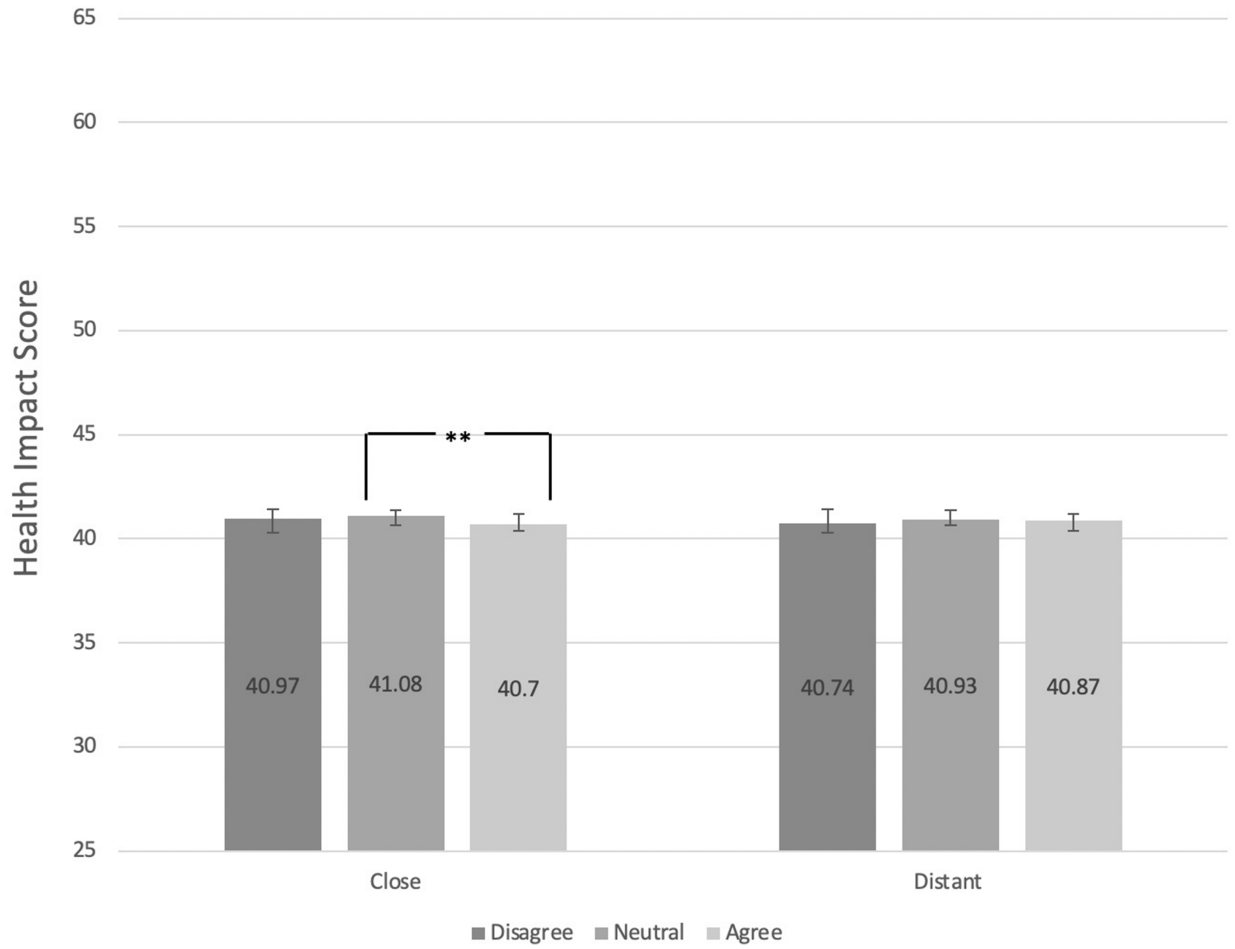
Mean environmental scores (possible range 0–100, range of sample 26–81; lowest to highest impact) of participant shopping baskets by level of agreement with statement suggesting environmentally friendly food choice social norms among their close and distant referent groups.

For this analysis, social norm perception scores were collapsed into three levels: Disagree (combining “Strongly disagree” and “Disagree”), Neither agree nor disagree, and Agree (combining “Strongly agree” and “agree”). Kruskal-Wallis tests indicated that there was no association between the perceptions of norms referring to the distant referent group and environmental scores (χ^2^ = 1.77, *p* > 0.05). However, pairwise comparisons using Dunn’s tests following significant Kruskal-Wallis test results ((χ^2^ = 15.44, *p* < 0.001, η^2^ = 0.0054) indicated that participants who perceived making environmentally friendly food choices to be the norm among the close referent group had shopping baskets with lower environmental impact scores compared to those who disagreed with this norm (*p* < 0.001) and to those who were neutral (*p* < 0.01; Figure 3).

Those who agreed with the “environment, close” social norm item, had a 0.90-point (*p* < 0.01, 95% CIs: −1.49, −0.28) and 1.3-point (*p* < 0.001, 95% CIs: −1.92. -0.66) reduction in their environmental impact, in comparison to those who were neutral and disagreed with the item, respectively. This relationship remained statistically significant after intervention arm (environmental labels, health labels, both, neither) was included in the models as a covariate (agree vs. neutral 0.80-point decrease, *p* < 0.01, 95% CIs: −1.40, −21; agree vs. disagree: 1.2-point decrease, *p* < 0.001, 9%% CIs: −1.83. -0.57). For verification, environmental impact scores of those who disagreed with the close referent group social norm statement were also compared to those who were neutral, and no difference was found (0.40-point decrease in negative environmental impact, *p* > 0.05, 95% CIs: −0.97, 0.17). However, it should be noted that the regression models had very small effect sizes (Cohen’s f^2^ values for norm only model = 0.007, for norm and condition model = 0.005).

There was no evidence that those who agreed with the “environment, distant” social norm item had statistically significantly different shopping basket scores compared to those who were neutral (0.43-point decrease, *p* > 0.05, 95% CIs: −1.12, 0.25) and to those who disagreed (0.21-point decrease, *p* > 0.05, 95% CIs: −0.88, 0.48; see Table 3).

**Table 3:** Linear regression models for effects of environmental social norms and intervention condition on environmental impact of shopping baskets.

	Environment, Distant		Environment, Close	
Independent Variables	β	[CI] (95%)	β	[CI] (95%)
Model 1 (Norm only)				
Disagree Agree	−0.23 −0.43	[−0.77, 0.31] [−1.12, 0.25]	0.40 −0.90**	[−0.17, 0.97] [−1.49, −0.28]
Model 2 (Norm + Condition)				
Disagree Agree	−0.23 −0.39	[−0.77, 0.31] [−1.1, 0.29]	0.40 −0.80**	[−0.17, 0.97] [−1.40, −0.21]
Condition				
Eco Health Eco + Health	−1.53*** −0.39 −2.2***	[−2.38, −0.68] [−1.2, 0.43] [−3.04, −1.33]	−1.46*** −0.36 −2.14***	[−2.3, −0.61] [−1.2, 0.46] [−2.99, −1.28]
R^2^ (M1) (M2)	0.0007 0.0182		0.0068 0.0235	
F^2^ (M1) (M2)	0.007 0.0		0.0068 0.005	
*F*(2, 2,478) (M1) *F*(5,2,475) (M2)	0.84 9.22		8.09 11.07	

Statistically significant beta values have been highlighted in bold. * = *p* < 0.05, ** = *p* < 0.01, and *** = *p* < 0.001.

### Effects of perceptions of social norms on shopping basket health scores

**Figure 4:**
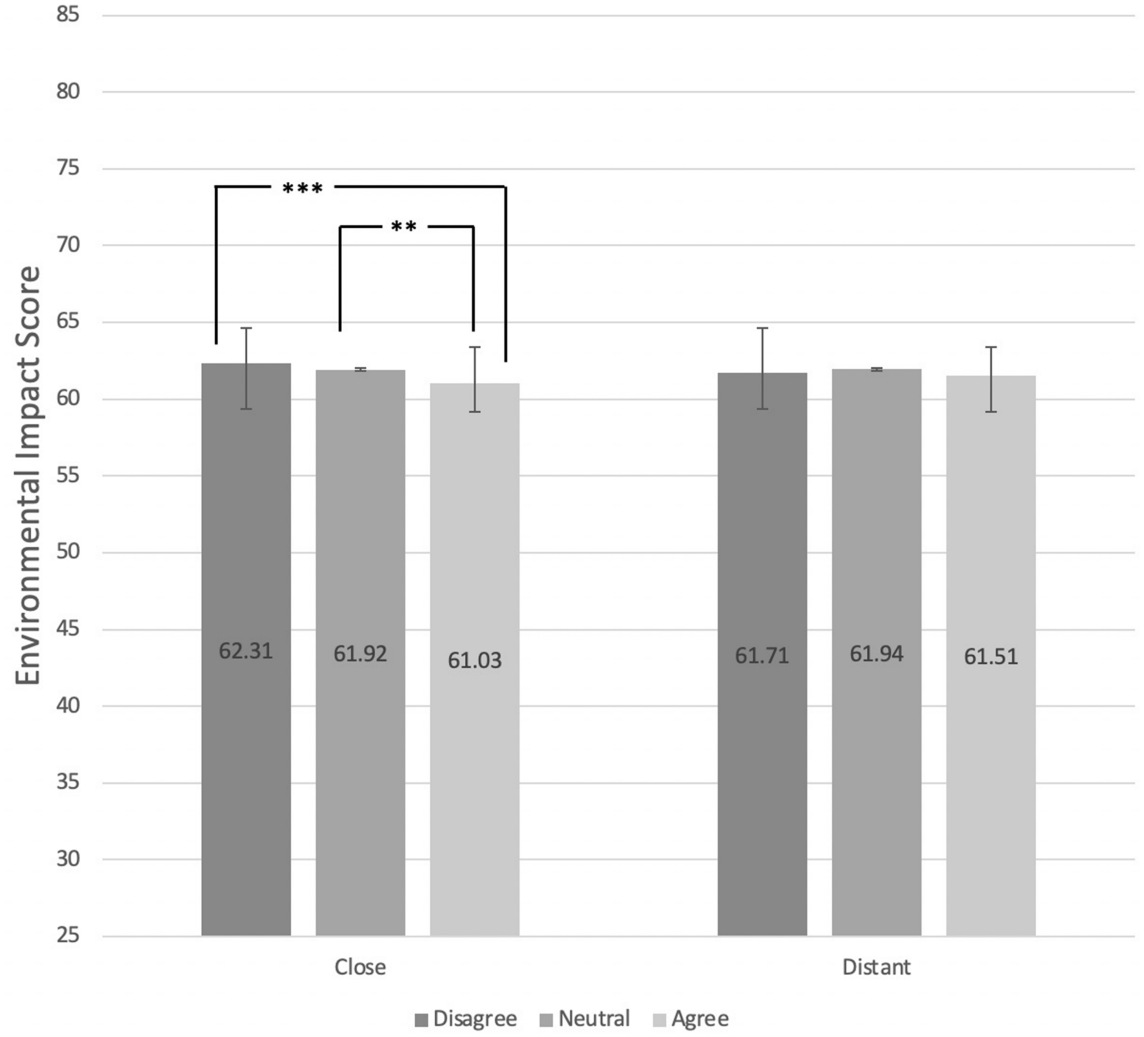
Mean health scores (possible range 0–100, range of sample 28–63; best to worst health impact) of participant shopping baskets by level of agreement with statement suggesting healthy food choice social norms among their close and distant referent groups.

As above, for this analysis, social norm perception scores were collapsed into three levels: Disagree (combining “Strongly disagree” and “Disagree”), Neither agree nor disagree, and Agree (combining “Strongly agree” and “agree”). Similar to the environmental scores, Kruskal-Wallis tests indicated that there was no association between the perceptions of norms referring to the distant referent group and health scores (χ^2^ = 0.17, *p* > 0.05). However, pairwise comparisons using Dunn’s tests following significant Kruskal-Wallis test results (χ^2^ = 8.37, *p* < 0.01, η^2^ = 0.0026) indicated that participants who perceived making environmentally friendly food choices to be the norm among the close referent group had shopping baskets with lower environmental impact scores compared to) and to those who were neutral (p < 0.01). Interestingly, no differences were found between those who agreed with the close referent group health norm and those disagreed (Figure 4).

Those who agreed with the “health, close” social norm item had a 0.38-point reduction (*p* < 0.05, 95% CIs: −0.68, −0.08) in their health impact compared to those who were neutral. This relationship remained statistically significant after intervention arm (environmental labels, health labels, both, neither) was included in the model as a covariate statement (0.38-point decrease *p* < 0.05, 95% CIs: −0.68, −0.07). Contrastingly, there was not a statistically significant reduction of shopping basket health impact scores when comparing those who agreed with the item to those who disagreed (0.27-point decrease, *p* > 0.05, 95% CIs: −0.68, 0.13). For verification, health impact scores of those who disagreed with the item were also compared to those who were neutral, and no difference was found (0.10-point increase in negative health impact, *p* > 0.05, 95% CIs: −0.33, 0.53). However, it should be noted that the regression models had very small effect sizes (Cohen’s f^2^ values for norm only model = 0.0031, for norm and condition model = 0.000).

There was no evidence that those who agreed with the “health, distant” social norm item had statistically significantly different shopping basket scores compared to those who were neutral (0.06-point decrease, *p* > 0.05, 95% CIs: −0.37, 0.25) and to those who disagreed (0.13-point increase, *p* > 0.05, 95% CIs: −0.21, 0.47; see Table 4).

**Table 4:** Linear Regression Models for effects of health social norms and intervention condition on health impact of shopping baskets.

	Health, Distant		Health, Close	
Independent variables	β	[CI] (95%)	β	[CI] (95%)
Model 1 (Norm only)				
Disagree Agree	−0.19 −0.06	[−0.54, 0.16] [−0.37, 0.25]	−0.10 −0.38*	[−0.53, 0.33] [−0.68, −0.08]
Model 2 (Norm + Condition)				
Disagree Agree	−0.19 −0.06	[−0.54–0.16] [−0.37, 0.25]	−0.10 −0.38*	[−0.53, 0.34] [−0.68, −0.07]
Condition				
Eco Health Eco + Health	0.02 −0.07 −0.16	[−0.44, 0.49] [−54, 0.41] [−0.62, 0.30]	0.02 −0.07 −0.16	[−0.46, 0.50] [−0.55, 0.41] [−0.66, 0.32]
R^2^ (M1) (M2)	0.0005 0.0009		0.0026 0.0	
F^2^ (M1) (M2)	0.0009 0.0		0.0026 0.0021	
F(2, 2,478) (M1) F(5,2,475) (M2)	0.58 0.46		3.27 1.52	

## Discussion

This study aimed to investigate the role of referent groups in perceptions of social norms regarding healthy and environmentally friendly food choices. Trying to make healthier food choices was perceived to be the norm more than trying to make environmentally friendly ones. This result is consistent with recent findings of a Healthy and Sustainable Diets Consumer Poll conducted by the Food Standards Agency of the UK (Heard and Bogdan, 2021), where compared to 87% of people saying eating a healthy diet was important to them, only 73% thought that eating sustainably was important. Similarly, while 75% reported that they knew what a healthy diet should consist of, this number dropped to 48% when asked about knowledge of what a sustainable diet should consist of. This comparative lack of awareness and prioritization of sustainable diets compared to healthy ones could explain why our sample also perceived both their distant and close referent groups to be trying to make healthier choices more than environmentally friendly ones. Individuals perceived people who they share their meals with to be trying to make more environmentally friendly and healthier food choices when compared to the general UK population. Going back to Social Identity Theory (Tajfel et al., 1979), this finding could be explained by the fact that individuals tend to see their in-groups (i.e., people they share meals with in this case) in a more positive light and attribute more positive behaviors to them (i.e., trying to make healthier and more environmentally friendly food choices) compared to the outgroup (i.e., other people in the UK).

The perception of stronger social norms among participants’ close referent groups to try and make environmentally friendly and healthy food choices was positively associated with participants’ selection of food products that had less negative environmental and health impacts in the online shopping task, demonstrating a consistency of a relationship between the perceived norm and individual behavior which persisted across different intervention arms of the labelling study. This relationship was not found for either environmental or health norms in the distant referent group. These findings provide further evidence that the use of different referent groups can change perceptions of social norms around food choices. Moreover, they suggest that perceived norms in a closer, more socially relevant group that the individual is more likely to identify with, can influence related behaviors more than norms that refer to a general social group. However, it should be noted that the nature of the study and its analyses were simply associational; while we interpreted our findings to suggest that perceived norms around healthy and environmentally food choices to be affecting participants’ behavior in the shopping task, a reverse relationship could also be possible. Individuals who already engage in sustainable and healthy behaviors may have a confirmation bias and be more perceptive of others acting in a similar manner, and thus think that these behaviors constitute the norm.

Our findings are consistent with previous findings that referent groups can moderate the perceived prevalence of social norms and their influence on healthy eating behavior, increasing fruit and vegetable intake, and reducing meat consumption (Cruwys et al., 2012; Stok et al., 2014; Higgs et al., 2019; Liu et al., 2019; Sparkman et al., 2020). The moderating role of referent groups have also been demonstrated in behavioral intervention studies. For example, a series of studies conducted in university restaurants in the United States found dynamic descriptive norm messages to be effective in promoting a shift from animal to plant-based foods when customers had “a greater connection to the norm referent” used in the messaging intervention (Sparkman and Walton, 2017). While a subsequent study in the United Kingdom failed to reproduce these results, it has only used a general referent group (“people in the UK”) and did not include a measure of in-group identification with the referent, which the authors have acknowledged as a limitation that warrants further study (Aldoh et al., 2021). Echoing this, another study that has tested a dynamic norm messaging intervention in restaurants across the UK also did not find evidence of the intervention changing plant-based food sales, noting that the vague referent group of “other customers” may not have been socially relevant for restaurant goers to identify with and create a desire to conform to their behavior (Çoker et al., 2022). Somewhat contrastingly, Thomas et al. (2017) have found that using a non-specific referent group (“most people”) norm message has been effective in increasing vegetable intake in worksite restaurants, but have argued that the familiarity with coworkers that restaurant goers had could have motivated them to conform to the norm and model their behavior. While messages with general referent groups might be ineffective in motivating norm conformity in restaurants where consumers go very infrequently and do not know or recognize others, these might be sufficient in settings where people are exposed to the norm message repeatedly and are also more likely to dine with a similar subgroup of people (e.g., coworkers) every day, making the observed behavior come from a socially close referent group. These limited findings from social norm intervention studies suggest that further research is necessary to understand the moderating role of referent groups in the efficacy of interventions. Previous research that looked at the role of perception of norms on meat consumption and other pro-environmental behaviors without trialing an intervention has found evidence that perception of descriptive and injunctive norms that refer to family, friends and significant others can be associated with self-reported behaviors, however it is worth noting that the study designs had some limitations. For example, both Sharps et al. (2021) and Culiberg and Elgaaied-Gambier (2016) had small and non-representative samples (136; 246 and 215, respectively) and consisting of 81% females in the case of the former and entirely of university students in the latter, and relied on self-reporting of behaviors.

The present study benefited from a large sample of 2,730 respondents, with 2,481 included in the final analyses with only a 9.12% attrition rate, that were representative of the general UK population in terms of gender, age, education and income, a rare and valuable opportunity in social norm and eating behavior research. Instead of relying on self-reported intentions, the study used an online shopping task using a virtual supermarket platform that was designed to strongly resemble a familiar online supermarket website with a similar and wide range of food products available to choose and organized across similar categories, which made the task a robust proxy for actual food purchase behavior. The shopping task also measured food selections across a variety of categories including fruits and vegetables, dairy, meat and confectionery. While the majority of social norm research around eating behaviors has examined fruit and vegetable intake, by focusing on both the environmental and health impacts of food choices across a variety of food products, the present study highlights the relationship between perceived concerns about sustainability or health and broader diet.

We measured the perception of the presence of norms around considering environmental and health impacts of items when making food choices. However, we did not ask individuals to rate the importance of these norms, i.e., how much these norms matter to them and how much they wish to conform to the behaviors the norm prescribes. The measure for behavior was also a proxy which was assessed through a virtual shopping task, where individuals did not spend real money, nor did they need to purchase or consume real food. Although the study had a representative sample for the UK in terms of age, gender, education, and income, it did not include any quotas for ethnic subpopulations. Due to the intrinsically cultural and social nature of eating behaviors, understanding how relevant norms are shaped across different ethnic groups and subpopulations may prove crucial for designing effective social norm interventions to promote more sustainable food choices within different contexts. People who identify as vegetarian and vegan were excluded from the sample, since the main study that preceded this survey also had an online shopping task component that required “purchase” of meat and dairy products. This meant that we were not able to capture the perceptions of social norms around healthy and environmentally friendly food choices of these subpopulations. It should also be noted that although we have found significant differences in perceptions of social norms around environmentally friendly versus healthy food choices and norms referring to distant versus close referent groups, the effect sizes were considerably small. Likewise, the regression models that examined the effect of perception of norms on individual shopping baskets also had considerably small effect sizes. Therefore, the findings reported here should be interpreted with caution.

In summary, this study provides a clear snapshot of perceptions of norms for both environmentally friendly and for healthy food choices within a large sample, providing evidence for the importance of referent groups in the perception of social norms. It also provides evidence that there is a small yet significant difference in the environmental and health impacts of food choices participants make in a virtual shopping task based on whether they agree making environmentally friendly and healthy food choices is the norm among people who they share their meals with or not. Future interventions that leverage social norms to encourage more sustainable and healthy food choices in food choice environments such as workplace cafeterias or school and university dining could consider framing norm messages to refer to more specific referent groups such as a particular workforce or group of students that are socially close and relevant to the target population to increase their efficacy.

## Data availability statement

The datasets presented in this study can be found in online repositories. The names of the repository/repositories and accession number(s) can be found at: https://osf.io/7jxfq/.

## Ethics statement

The studies involving human participants were reviewed and approved by University of Oxford Central University Research Ethics Committee (Approval Reference: R65010/RE004). The patients/participants provided their written informed consent to participate in this study.

## Author contributions

EÇ designed and ran the study, conducted the data analysis, and wrote the manuscript. SJ supervised EÇ in the development, conducting and analysis of the study and was involved in the manuscript drafting, providing extensive feedback and edits. CS was involved in the development and conducting and analysis of the labeling study associated with the present study and provided feedback and edits on the manuscript. MC was involved in the data extraction and analysis of the food products used in the online supermarket task and the calculation of their environmental impact and nutritional values and provided feedback and edits on the manuscript. RP supervised EÇ in the development, conducting and analysis of the study, provided extensive feedback and edits on the manuscript, and was involved in the data collection, analysis and manuscript drafting of the labeling study associated with the present study. All authors contributed to the article and approved the submitted version.

## Funding

This research was funded by the Wellcome Trust, Our Planet Our Health (Livestock, Environment and People –LEAP) award number 205212/Z/16/Z.

## Acknowledgments

The authors would like to thank Kerstin Frie for her early supervision over the initial development of the study and data analysis plan and Christina Potter and Brian Cook for their valuable work in developing, conducting, analyzing, and writing up the manuscript for the food labeling intervention study that prepared the groundwork for the present study.

## Conflict of interest

The authors declare that the research was conducted in the absence of any commercial or financial relationships that could be construed as a potential conflict of interest.

## Publisher’s note

All claims expressed in this article are solely those of the authors and do not necessarily represent those of their affiliated organizations, or those of the publisher, the editors and the reviewers. Any product that may be evaluated in this article, or claim that may be made by its manufacturer, is not guaranteed or endorsed by the publisher.
